# IL-33 is produced by colon fibroblasts and differentially regulated in acute and chronic murine colitis

**DOI:** 10.1038/s41598-021-89119-1

**Published:** 2021-05-05

**Authors:** Amanda Waddell, Jefferson E. Vallance, Sejal Fox, Michael J. Rosen

**Affiliations:** 1 Division of Gastroenterology, Hepatology and Nutrition, Cincinnati Children’s Hospital Medical Center, 3333 Burnet Ave, MLC 2010, Cincinnati, OH 45229 USA; 2 Department of Pediatrics, University of Cincinnati College of Medicine, Cincinnati, OH USA

**Keywords:** Cytokines, Ulcerative colitis

## Abstract

IL-33 is upregulated in ulcerative colitis and has a protective role in chemically-induced acute murine colitis. We aimed to determine whether IL-33 influences *Il10*^−/−^ chronic colitis and its cellular source in health and during colitis. *Il10*^−/−^*Il33*^−/−^ and *Il10*^−/−^*Il33*^+/+^ littermates developed colitis of similar severity. Colon *Il33* was induced in WT and *Il10*^−/−^ mice exposed to DSS, but not in unchallenged *Il10*^−/−^ mice with colitis. *Il33*-citrine reporter mice showed that *Il33-*citrine colocalized with α-smooth muscle actin^+^ myofibroblasts and vimentin^+^ fibroblasts in WT mice. Citrine^+^CD74^+^CD90^hi^ inflammatory fibroblasts were increased with DSS treatment. IL-1β induced *Il33* expression in colon myofibroblasts, but colon *Il33* expression did not differ between DSS-treated WT and *Il1r1*^−/−^ mice. In conclusion, deficiency of IL-33 does not alter the severity of chronic colitis in *Il10*^−/−^ mice. Induction of *Il33* upon DSS exposure in WT and *Il10*^−/−^ mice, but not in unchallenged *Il10*^−/−^ mice, suggests epithelial injury induces colon IL-33. Fibroblasts are the primary colonic source of IL-33 and IL-33-expressing CD90^hi^CD74^+^ fibroblasts are increased during DSS-induced colitis. IL-1β induces *Il33* in colon myofibroblasts in vitro, but signaling through the IL-1R1 is not necessary for induction of IL-33 in DSS-induced colitis.

## Introduction

Inflammatory bowel disease (IBD), including ulcerative colitis (UC) and Crohn’s disease, is a group of disorders of chronic intestinal inflammation and progressive bowel damage caused by a complex interplay of genetic, microbial and environmental factors^1^. Cytokines are central to IBD pathogenesis, with roles for both driving and controlling the mucosal inflammation^2^. Advances in our understanding of cytokine biology have led to the development of transformative therapies for IBD. Still, 40 and 70% of UC patients will not achieve mucosal healing with anti-TNF therapy and anti-IL12/23 therapy, respectively, and 70% of available anti-cytokine biologic drugs frequently fail to heal the mucosa^3,4^. Defining new mechanisms for intestinal inflammation is a clear need for developing new effective therapies in IBD.

IL-33, a member of the IL-1 superfamily, is increased in patients with UC and a polymorphism has been associated with UC and an extensive colitis phenotype^5–11^. IL-33 is often described as an alarmin released by epithelial and endothelial cells in the setting of injury, but it is also expressed by innate immune cells^12–14^. In the intestine, previous studies have shown that IL-33 is expressed in subepithelial fibroblasts, but further phenotyping has not been done^15,16^. Our lab has previously identified a protective role for IL-33 in oxazolone colitis, through protecting epithelial cells, particularly goblet cells^17^, which is mediated by ILC2-derived IL-13^18^. Other studies have also shown that IL-33 has a protective role by inducing epithelial-derived miR-320 that promotes epithelial repair and the resolution of inflammation^19^ and decreasing the pathogenic Th17 response in chronic DSS colitis^20,21^. Of the studies done on chronic models of colitis, only the role of exogenous IL-33 was explored^22,23^, and exogenous IL-33 is known to effect intestinal physiology through goblet cell hypertrophy and hyperplasia^5^. However, studies have not examined the role of endogenous IL-33 in a chronic model of colitis.

The role for endogenous IL-33 in chronic colitis as well as regulation of IL-33 expression remains uncertain. In our study, we examine differences in the role of IL-33 in chronic vs. acute colitis. Interestingly, IL-33 is strongly induced during DSS-induced colitis, but not during IL-10^–/–^ chronic colitis. Although a protective role has been shown for IL-33 in other models of colitis, we could find no differences in colitis development or severity in *Il10*^−/−^ compared to *Il10*^−/−^*Il33*^−/−^ mice. Using IL-33-citrine reporter mice (*Il33*^*Cit/*+^), we show that vimentin^+^CD90^+^ fibroblasts are the source of IL-33 at baseline and this population increases in DSS-induced colitis. Although IL-1β induced IL-33 expression in vitro, using *Il1r1*^−/−^ mice, we showed this signaling pathway is not required for *Il33* induction during DSS-induced colitis. Interestingly, CD90^+^CD74^+^ inflammatory fibroblasts express IL-33 and are increased in DSS-induced colitis, indicating this population is likely responsible for the increases in *Il33* in acute colitis induced by epithelial damage.

## Results

### IL-33 deletion does not alter course of **Il10**^−/−^ colitis

**Figure 1:**
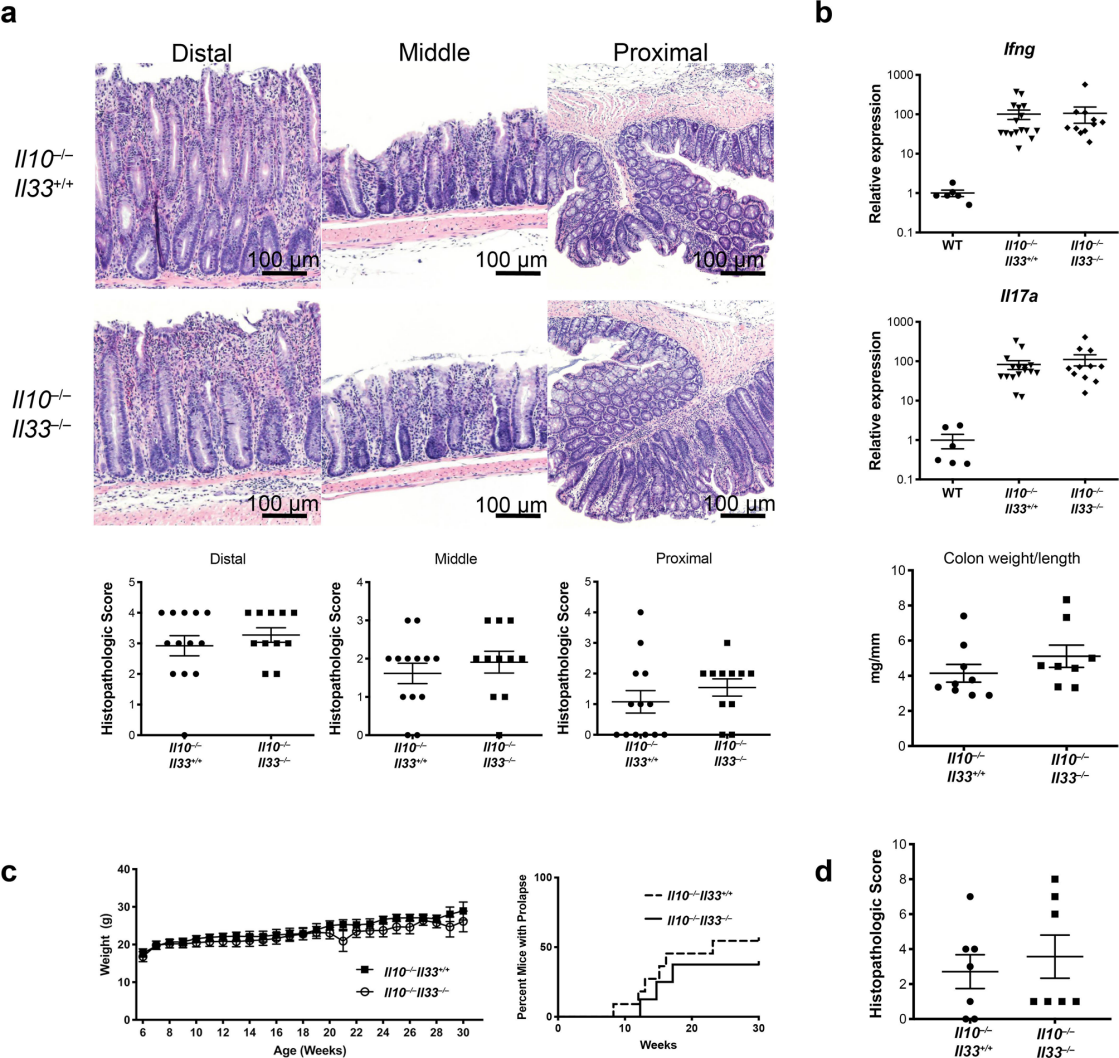
IL-33 deletion did not alter severity of *Il10*^−/−^ colitis. (**A**) Representative H&E sections from 12-week old *Il10*^−/−^*Il33*^+/+^ and *Il10*^−/−^*Il33*^−/−^ mice and histological scoring. (**B**) RT PCR analysis at 12 weeks old. (**C**) Weight change and prolapse occurrence up to 30 weeks old. (**D**) Histopathological scores of 30-week old *Il10*^−/−^*Il33*^+/+^ and *Il10*^−/−^*Il33*^−/−^ mice. N = 7–16/group.

To determine the role of IL-33 in a spontaneous chronic model of colitis, we crossed *Il10*^−/−^ mice to *Il33*^−/−^ to generate *Il10*^−/−^ *Il33*^−/−^ mice. *Il10*^−/−^*Il33*^−/−^ and *Il10*^−/−^*Il33*^+/+^ littermates developed colitis of similar severity beginning at approximately 12 weeks of age. There were no differences in histopathologic severity between the two strains (Fig. 1A). Tissue *Ifng* and *Il17a*, two T cell cytokines known to be involved in disease, were examined in the two strains and both were induced similarly in *Il10*^−/−^*Il33*^−/−^ and *Il10*^−/−^*Il33*^+/+^ compared to WT mice (Fig. 1B). We examined weight loss and rectal prolapse occurrence in both strains of mice through 30 weeks of age and found no differences (Fig. 1C). Histopathological scores were also not different at 30 weeks of age (Fig. 1D). Collectively this data does not support a role for IL-33 in regulating spontaneous chronic colitis in *Il10*^−/−^ mice.

### **Il33** is induced in DSS colitis but not **Il10**^−/−^ colitis

**Figure 2:**
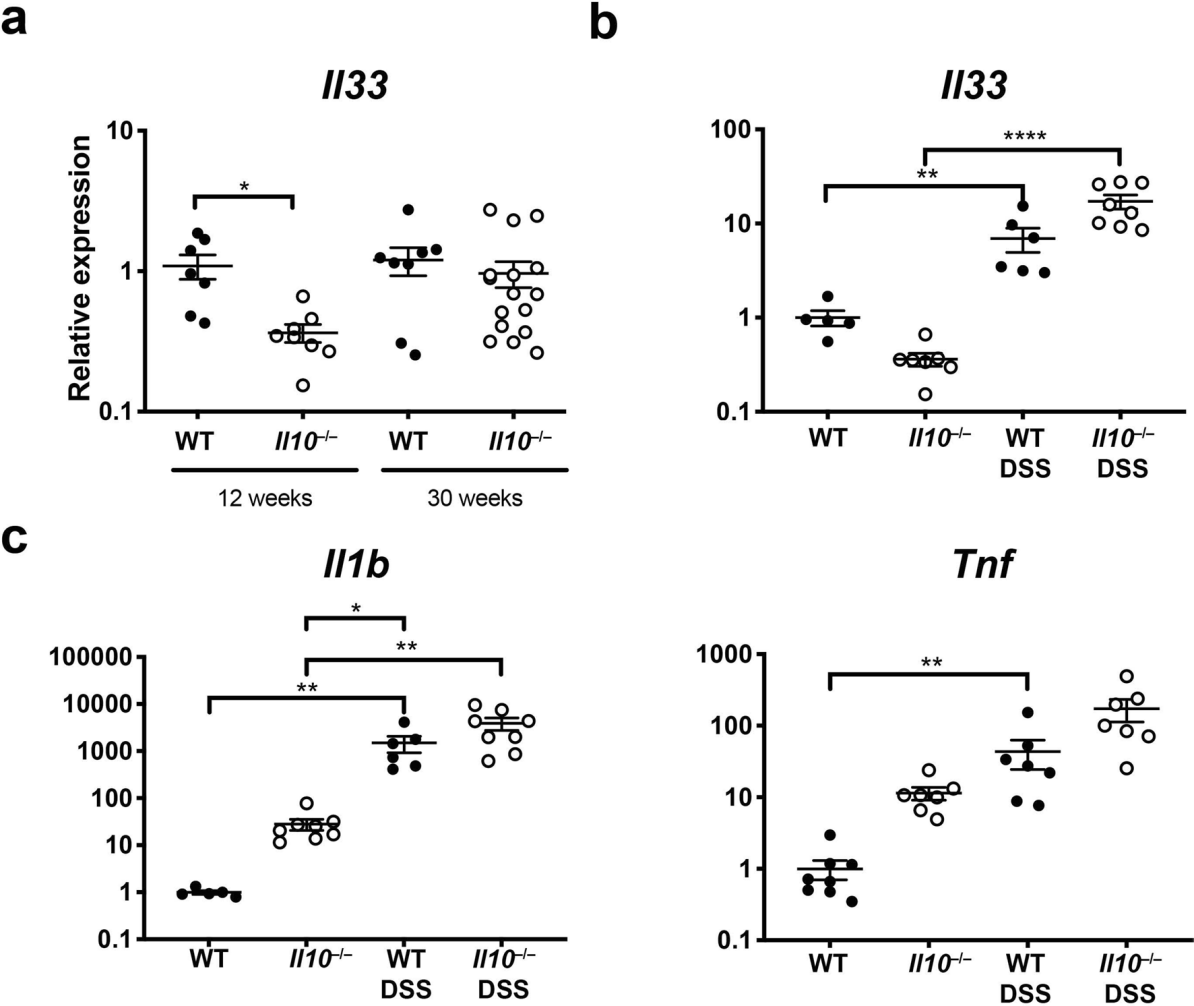
IL-33 was induced in acute DSS colitis but not chronic *Il10*^−/−^ colitis. *Il33* expression assessed by real-time RT-PCR in the distal colon of (**A**) 12-week- and 30-week-old mice WT and *Il10*^−/−^ mice or (**B**) 12-week old WT and *Il10*^−/−^ mice treated with DSS for 5 days and harvested on day 7. (**C**) *Il1b* and *Tnf* expression in *Il10*^−/−^ and WT mice with or without DSS treatment. N = 5–8/group **p < 0.01, ****p < 0.0001.

To better understand the lack of an effect of *Il33* deficiency on colitis in *Il10*^−/−^ mice, we examined colon *Il33* expression in WT and *Il10*^−/−^ mice at 12 and 30 weeks of age. *Il33* was not increased in *Il10*^−/−^ mice with chronic colitis compared to WT mice (Fig. 2A). Others have demonstrated a protective role for IL-33 in DSS-induced colitis^19,24,25^. In contrast to the chronic *Il10*-deficient model, we observed a sevenfold increase in colon *Il33* expression in acute colitis induced by epithelial injury from DSS compared to untreated WT mice (Fig. 2B), indicating that factors specific to DSS colitis may be regulating IL-33. To determine whether IL-10 is required for induction of IL-33 during colitis, we exposed *Il10*^−/−^ mice to DSS. DSS exposure induced colon *Il33* expression significantly compared to untreated mice (Fig. 2B), suggesting that IL-10 is not required for *Il33* induction during colitis.

To begin to understand what difference might be leading to differences in regulation of IL-33 in the two models of murine colitis, we examined tissue RNA expression for two cytokines that have previously been shown to induce IL-33, TNF and IL1-β^16^. Colon *Tnf* expression was 4.6 fold higher, and, strikingly, *Il1b* expression was 1419-fold higher in DSS-treated WT mice compared to 12-week old IL-10KO mice with colitis (Fig. 2C).

### Colon fibroblasts are the main source of *Il33*

**Figure 3:**
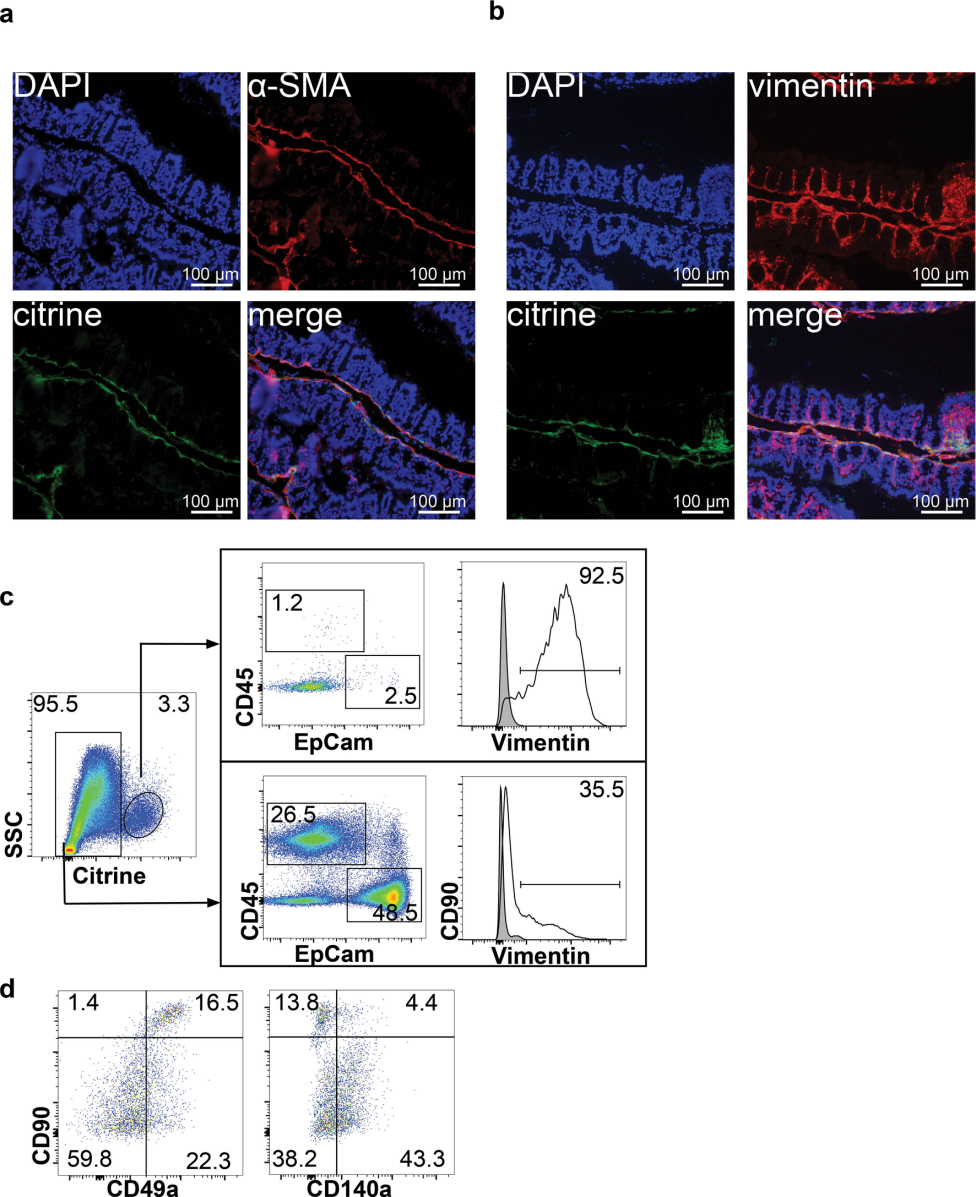
Fibroblasts were the primary source of IL-33 in the healthy mouse colon. Representative immunofluorescence photomicrographs of proximal colon from *Il33*^*Cit/*+^ reporter mice co-stained with (**A**) α-SMA for myofibroblasts and (**B**) vimentin for total fibroblasts. (**C**) Flow cytometric analysis of colon lamina propria cells that express citrine in healthy *Il33*^*Cit*/+^ mice. Shaded histogram is fluorescence minus one control. (**D**) Flow cytometry for fibroblast markers in citrine^+^ cells. Representative flow plots from 3 independent experiments.

IL-33 citrine reporter mice (*Il33*^*Cit*/+^) were used to investigate the cellular source of IL-33 in unchallenged WT mice. This reporter uses citrine fluorescence as a surrogate for IL-33 mRNA expression with the GFP-derived Citrine gene inserted directly downstream of the ATG start codon of *Il33*^14^. Immunofluorescence microscopy colocalized citrine expression in the proximal colon with alpha smooth muscle actin (α-SMA) and vimentin in unchallenged *Il33*^*Cit*/+^ mice, suggesting IL-33 is expressed by colon myofibroblasts (Fig. 3A,B). To confirm this, flow cytometry was performed for epithelial, hematopoietic and fibroblast markers on the lamina propria fraction. In the lamina propria, citrine^+^ cells were EpCam^−^, CD45^−^, and > 85% vimentin^+^, consistent with fibroblasts (Fig. 3C).

Fibroblasts are a complex population of many different subsets. As single cell studies have demonstrated, stromal cells can be subdivided into as many as six subsets^26^. To further phenotype these cells, we also examined expression of CD90, CD140a and CD49a (Fig. 3D). CD90 is reported to be expressed on fibroblasts located in close proximity to stem cells and support organoid growth in vitro^27^. CD49a is α1 integrin and is involved in fibroblast proliferation and adhesion to collagen^28^. CD140a is PDGFRA and is considered a pan-fibroblast marker and CD140a^+^ cells are important for maintaining the intestinal stem cell niche^29^. The IL-33-expressing population of fibroblasts was heterogeneous, with CD90^hi^, CD90^mid^ and CD90^−^ cell populations. The citrine^+^CD90^hi^ fibroblast population expressed CD49a, but the majority of these cells were CD140a^−^ (Fig. 3D).

### IL-1β induced *Il33* expression in colon fibroblasts

**Figure 4:**
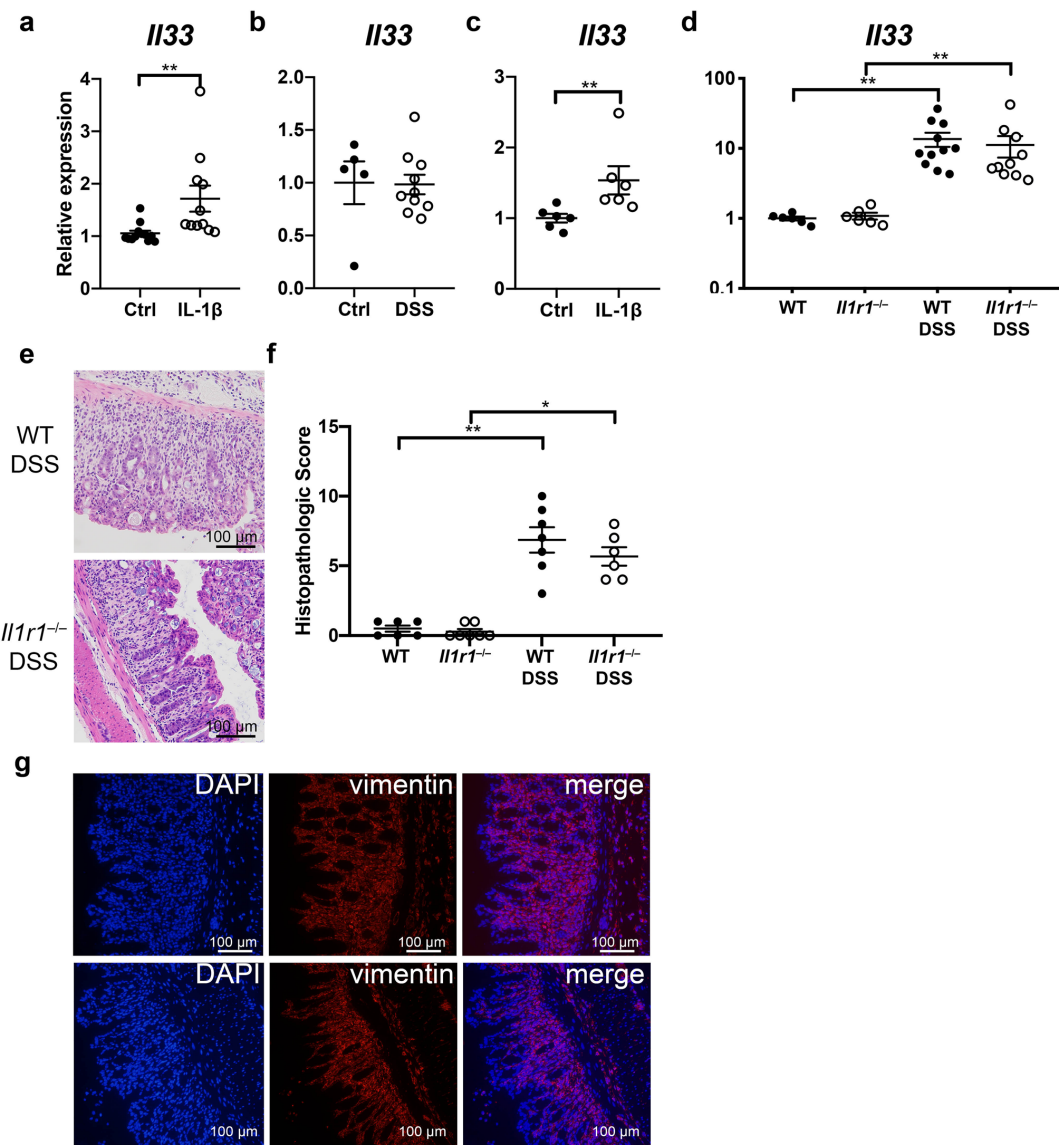
IL-1β induced *Il33* expression in colon fibroblasts in vitro. *Il33* expression assessed by real-time RT-PCR in (**A**) IL-1β treated and untreated colon myofibroblast WEHI-YH2 cells from 3 independent experiments and (**B**) Citrine^+^ cells sorted from Control and DSS-treated *Il33*^*Cit*/+^ mice. (**C**) Citrine^+^ cells sorted from DSS-treated *Il33*^*Cit*/+^ mice stimulated with IL-1β, and (**D**) colon tissue from WT and *Il1r1*^−/−^ mice given DSS. (**E**) Representative H&E staining and (**F**) histopathologic scores of WT and *Il1r1*^−/−^ mice treated with DSS. (**G**) Representative images of vimentin immunofluorescent staining. N = 5–10 mice/group, *p < 0.05, **p < 0.01.

Since we observed substantial differences in the induction of *Il1b* between colitis induced by DSS and IL-10-deficiency, we sought to determine whether IL-1β regulates *Il33* expression in colon myofibroblasts, as has been reported by others^15^. The WEHI-YH2 colon myofibroblast cell line^30^ was stimulated with IL-1β for 18 h and *Il33* expression was analyzed. IL-1β increased *Il33* in WEHI cells compared to control (Fig. 4A). To examine primary cells, citrine^+^ cells were sorted from control and DSS-treated mice. Although *Il33* was not increased in unstimulated citrine^+^ cells from DSS-treated mice compared to those from control mice (Fig. 4B), ex vivo stimulation with IL-1β increased *Il33* expression in citrine^+^ cells from DSS-treated mice (Fig. 4C), indicating that IL-1β could play a role in the regulation of IL-33 in the colon during colitis.

To examine the role of IL-1β in vivo, we treated WT and *Il1r1*^−/−^ mice deficient for the IL-1β receptor with DSS and examined colon *Il33* expression. We found no difference in *Il33* induction between WT and *Il1r1*^−/−^ mice treated with DSS (Fig. 4D). WT and *Il1r1*^−/−^ mice treated with DSS exhibited similar histopathologic severity (Fig. 4E,F). Although *Il33* expression was induced with DSS in WT and *Il1r1*^−/−^ mice, expression did not correlate with histopathologic severity in either strain (WT, Spearman r 0.61, p = 0.16; *Il1r1*^−*/*−^, Spearman r 0.03, p > 0.99)*.* Furthermore, immunofluorescence staining for vimentin indicated a similar number of fibroblasts between WT and *Il1r1*^−/−^ mice treated with DSS (Fig. 4G). These data indicate that IL1R1 signaling is not required for the induction of *Il33* in the colon during DSS-induced colitis in vivo.

### IL-33-citrine^+^ fibroblasts are increased during DSS, but not IL-10-deficient colitis

**Figure 5:**
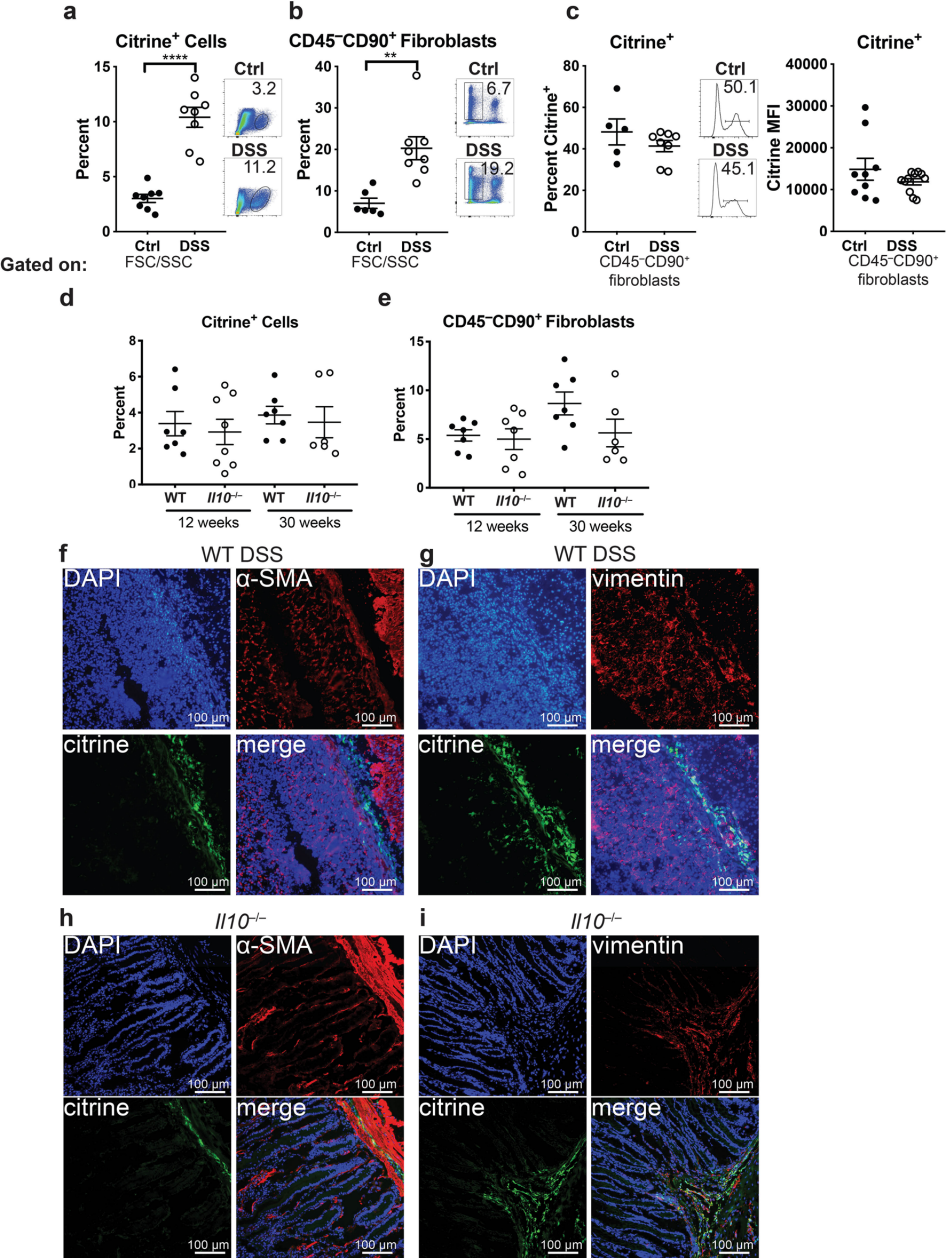
Increased colon mucosal fibroblasts in colitis induced by DSS but not IL-10-deficiency. Comparisons of percent of colon lamina propria cells that are (**A**) citrine^+^ *ll33-*expressing cells or (**B**) CD45^−^CD90^+^ fibroblasts, and (**C**) percent of lamina propria citrine^+^ *ll33-*expressing fibroblasts and citrine MFI in unchallenged and DSS-treated *Il33*^*Cit*/+^ mice. Comparisons of percent of colon lamina propria cells that are (**D**) citrine^+^ *ll33-*expressing cells or (**E**) CD45^−^CD90^+^ fibroblasts in 12- and 30-week old WT and *Il10*^−/−^ mice. Representative immunofluorescent images from DSS-treated *Il33*^*Cit*/+^mice (**F**) and (**G**) and *Il10*^−/−^ *Il33*^*Cit*/+^mice (**H**) and (**I**) N = 5–8 mice/group **p < 0.01, ****p < 0.0001.

Since *Il33* expression increases in DSS-induced colitis, we examined IL-33-citrine^+^ cells in DSS-exposed and unchallenged *Il33*^*Cit*/+^mice by flow cytometry. Over 90 percent of Citrine^+^ cells in DSS-induced colitis and *IL10*^−/−^ colitis were vimentin^+^ fibroblasts. Approximately 5% were CD45^+^ leukocytes, most of which were F4/80^+^ macrophages (Supplementary Fig. 1). We went on to demonstrate that CD45^−^CD90^+^ cells correspond to vimentin^+^ cells, allowing us to use surface markers to identify fibroblasts (Supplementary Fig. 1). Citrine^+^ cells were significantly increased during DSS-induced colitis compared to unchallenged control mice (Fig. 5A). This increase in citrine^+^ cells was due to a generalized increase in CD45^−^CD90^+^ fibroblasts in the colon (Fig. 5B), but not due to a higher percentage of fibroblasts expressing IL-33 (Fig. 5C), indicating that both IL-33^+^ and IL-33^−^ fibroblasts are increasing during DSS-induced colitis. Furthermore, citrine mean fluorescence intensity (MFI) was not increased in CD45^−^CD90^+^ fibroblasts from DSS-treated mice compared to untreated mice, indicating that transcription of *Il33* is not increased on a per cell basis (Fig. 5C). To examine the source of IL-33 in *Il10*^−/−^ mice, we generated *Il10*^−/−^*Il33*^*Cit*/+^mice. In contrast to the DSS model, during colitis in 12-week or 30-week old *Il10*^−/−^*Il33*^*Cit*/+^ mice, neither citrine^+^ cells nor CD45^−^CD90^+^ fibroblasts were increased compared to littermate *Il10*^+/-^*Il33*^*Cit*/+^ mice (Fig. 5D,E). Immunofluorescence in DSS-treated *Il33*^*Cit*/+^mice (Fig. 5F,G) and *Il10*^−/−^*Il33*^*Cit*/+^ mice (Fig. 5H,I) demonstrated vimentin^+^citrine^+^ cells in areas of inflammation. Interestingly, these cells are α-SMA^−^, indicating these cells are different from the α-SMA^+^citrine^+^ cells seen in untreated mice (Fig. 3). These data support that increased *Il33* expression in DSS-induced colitis may be explained by an expanded population of *Il33*-expressing colon mucosal fibroblasts, proportional to an overall expansion of fibroblasts, which is not observed in *Il10*^−/−^ colitis.

**Figure 6:**
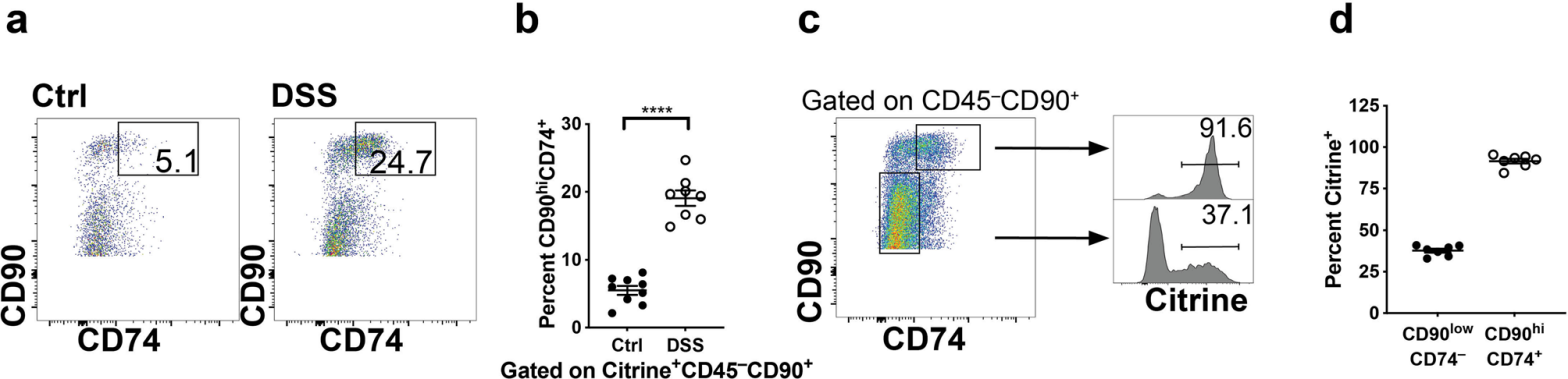
Citrine^+^ fibroblasts induced by DSS are CD90^hi^CD74^+^ inflammatory fibroblasts. (**A**) Flow cytometric analysis and (**B**) quantification of CD90^hi^CD74^+^ cells gated on citrine^+^CD45^−^CD90^+^ fibroblasts in the colon lamina propria at baseline and during DSS-induced colitis. (**C**) Flow cytometric analysis and (**D**) quantification of citrine^+^ cells gated on CD45^−^CD90^+^ fibroblasts to show which fibroblast population is mainly expressing IL-33-citrine during DSS. N = 8–9 mice/group ****p < 0.0001.

Recently single cell RNA-seq approaches have brought attention to an expanded population of inflammatory fibroblasts as a key signaling hub in UC^26,31^. To further phenotype the IL-33^+^ fibroblasts in DSS-induced colitis, we examined the inflammatory marker CD74, which has been shown to be expressed on inflammatory fibroblasts^26^. Inflammatory citrine^+^ CD90^hi^CD74^+^ fibroblasts were markedly increased in DSS-treated compared to unchallenged *Il33*^*Cit*/+^mice (Fig. 6A,B). Furthermore, > 90% of CD90^hi^CD74^+^ fibroblast are citrine^+^ while only 33–41% of CD90^low^CD74^−^ fibroblasts are citrine^+^ (Fig. 6C,D) demonstrating that CD90^hi^CD74^+^ fibroblasts have a greater capacity for making IL-33.

## Discussion

In the present study, we demonstrate that mucosal fibroblasts are the main cellular source of IL-33 in the mouse colon. During acute colitis induced with DSS, we show there is an expansion of IL-33-producing inflammatory fibroblasts similar to those reported in human UC. Surprisingly, we found that neither *Il33* expression, nor IL-33 producing fibroblasts were increased during the development of chronic colitis in *Il10*-deficient mice, although *Il33* expression could be induced in *Il10*^−/−^ mice by epithelial injury with DSS. Accordingly, using double knockout mice, we demonstrated that IL-33 does not play a significant role in the development or progression of chronic colitis in *Il10*^−/−^ mice. We found that colon mucosal *Il1b* expression was induced to a much higher degree in DSS-induced compared to *Il10*^−/−^ colitis, and that IL-1β induced *Il33* from colon fibroblasts in vivo, however IL1R signaling was not required for induction of *Il33* expression during DSS.

Many studies have explored the role of IL-33 in acute murine models of colitis or recovery from colitis, but there is a paucity of studies in models of chronic colitis. The role of exogenous IL-33 has been examined in chronic colitis^22,23^, but supra-physiologic levels of IL-33 have profound effects on intestinal morphology through induction of Th2 cytokines^5^. To determine the role of endogenous IL-33 in chronic colitis, we bred *Il10*^−/−^/*Il33*^−/−^ mice and compared them to littermate *Il10*^−/−^/*Il33*^+*/*+^ mice. Mice spontaneously began to develop colitis around 8 weeks old and some mice were followed for up to 30 weeks. We expected to find a protective role for IL-33 in chronic colitis due to its protective role in other models of colitis, including our previous study in oxazolone colitis^17,19,22,24,25^. Some studies have shown a protective role for IL-33 by acting directly on the intestinal epithelium^17,19^. However, other studies have shown that IL-33 acts on ST2^+^ regulatory T cells to induce their proliferation and suppressive function^32,33^. Since IL-10 from regulatory T cells is known to be important in preventing development of intestinal inflammation^34^, the lack of functional IL-10-producing regulatory T cells could have prevented IL-33 from ameliorating colitis in this model. On the other hand, this potential explanation does not explain the finding of unchanged colon *Il33* expression during colitis in *Il10*^−/−^ mice. The lack of increased *Il33* expression *Il10*^−/−^ mice with colitis stands in stark contrast to the increased *Il33* expression consistently observed in human UC^7–10,17^. It may be that the degree of epithelial injury in chronic *Il10*^−/−^ colitis is insufficient to induce tissue IL-33 given that colon tissue *Il33* expression could be induced with DSS treatment in *Il10*^−/−^ mice.

Since previous studies have shown a protective role for IL-33 in DSS-induced colitis, we compared *Il33* induction in *Il10*^−/−^ chronic colitis and DSS-induced acute colitis. We found that *Il33* is significantly increased in DSS-induced colitis, but not in *Il10*^−/−^ colitis. However, *Il33* can be induced in *Il10*^−/−^ mice by DSS, suggesting that epithelial damage triggers colon IL-33 expression and that IL-33 induction is not limited by IL-10 deficiency itself. We sought to determine what molecular differences between DSS and *Il10*^−/−^ colitis could lead to this difference in *Il33* induction. We firstly found that *Il1b* was induced over 1000-fold more in DSS compared to *Il10*^−/−^ colitis. Furthermore, IL-1β has been shown to induce *Il33* in dermal and ileal fibroblasts^15,16^. Although we demonstrated that IL-1β induced *Il33* expression in both a colon myofibroblast cell line and *Il33*-expressing citrine^+^ cells isolated from DSS-treated *Il33*^*Cit*/+^ mice in vitro, we did not observe any effect of loss of IL-1β signaling through its receptor on DSS-induced mucosal inflammation or *Il33* expression in vivo. Furthermore, our data in vivo suggest that an increase in the number of *Il33*-expressing fibroblasts, not fibroblast-intrinsic *Il33* expression, explains the overall increase in *ll33* in the colon. Therefore, although IL-1β may, in part, regulate colon fibroblast *Il33* expression, IL-1β signaling is not required for induction of *Il33* after epithelial injury with DSS. Further research is warranted to determine the factors that stimulate recruitment, differentiation, or proliferation of IL-33-producing colon fibroblasts by epithelial injury and or mucosal inflammation.

Others have demonstrated that intestinal IL-33 is also regulated by bacterial infection and colonization. Similar to the observed induction of IL-33 in DSS-induced colitis, colon IL-33 and IL-33 receptor are upregulated in acute infectious colitis with *Citrobacter rodentium.* In this infectious model, IL-33 drives mucosal inflammation and impairs bacterial clearance through enhancing epithelial permeability and limiting the induction of Th17 cells^35^. Stable colonization of mice with the Crohn’s disease-associated pathobiont adherent invasive *E. coli*, permitted only after colitis induced by *Salmonella* or DSS, also results in marked upregulation of IL-33 and the IL-33 receptor, which contributes to intestinal fibrosis in this model^36^. The sources of IL-33 have not been fully elucidated in these microbe-induced models.

Previous studies have shown that in the intestine, myofibroblasts and epithelial cells express IL-33 at baseline^8,15,16,37^. Using IL-33-citrine reporter mice^14^, we showed that the main cellular source of IL-33 in unchallenged mice and during acute colitis with DSS is CD90^+^vimentin^+^ fibroblasts. Immunofluorescent microscopy revealed that a subset of these cells were myofibroblasts expressing α-SMA. Further heterogeneity in this population is evidenced by populations of *Il33*-expressing citrine^+^ cells that are CD90^hi^CD49a^+^CD140a^−^ and CD90^mid^CD49a^−^CD140a^+/−^. Recent single cell RNA-seq studies of stromal cells in the intestine have shown diverse populations of fibroblasts in the colon of patients with UC^26,31^. Following DSS treatment, we observed an increase in citrine^+^ cells in DSS-treated mice compared to control mice, confirming an increase in *Il33*-expressing cells is leading to the increase in *Il33* during DSS.

We sought to determine which IL-33-citrine^+^ fibroblast population was increasing during DSS-induced colitis. One study found that IL-33-expressing colon fibroblasts also expressed the major histocompatibility complex class II invariant chain CD74 and these cells were enriched in active UC^26^. We found that CD90^hi^CD74^+^ cells expressing IL-33 were increased in DSS-treated mice compared to control mice. CD90 has previously been shown to be a marker for intestinal stromal cells that express IL-33 in response to *Salmonella typhimurium* and flagellin^27^. Furthermore, CD90^+^ fibroblasts have been shown to more strongly support epithelial growth compared to CD90^−^ fibroblasts using co-cultures with colonoids, resulting in significantly more colonoid budding and growth in co-cultures with CD90^+^ fibroblasts^38^. These studies support a protective, immunoregulatory role for this fibroblast population during colitis.

In conclusion, the primary source of IL-33 in the mouse colon, at baseline and during acute colitis induced by epithelial injury, is mucosal fibroblasts. The induction of tissue *Il33* expression after epithelial injury with DSS is related to an increase in *Il33*-expressing CD90^hi^CD74^+^ inflammatory fibroblasts. *Il33*-expressing fibroblasts are not induced during spontaneous chronic colitis in *Il10*^−/−^ mice and IL-33 deficiency does not affect colitis development or severity in *Il10*^−/−^ mice. IL-1β induces *Il33* expression from colon fibroblasts in vitro, but signaling through IL1R1 is dispensable for induction of *Il33* expression in colon tissue after epithelial injury with DSS in vivo. This study uncovers important cell and molecular differences between animal models of colitis and human UC with regard to cytokine expression and stromal cell populations. Future study of the action of IL-33-producing fibroblasts during colitis may uncover new pathways to translate for UC treatment.

## Materials and methods

### Mice and in vivo treatment

*Il10*^−/−^ (Jackson Labs strain 002251), *Il1r1*^−/−^ (Jackson Labs strain 003245), *Il33*^−/−^, *Il33-citrine* reporter (*Il33*^Cit/+^) and WT mice, all on the C57BL/6 background, were bred at CCHMC under specific-pathogen-free conditions and maintained on a standard laboratory chow diet in a half-day light cycle exposure and temperature-controlled environment. The generation of the *Il33*^−/−^ and *Il33*^Cit/+^ mice were previously described^14,39^. *Il10*^−/−^ and *Il33*^−/−^ strains were crossed to generate littermate *Il10*^−/−^*Il33*^−*/*−^ and *Il10*^−*/*−^*Il33*^+/+^ mice. *Il33*^Cit/+^ mice were crossed to *Il10*^−/−^ mice to generate *Il10*^−/−^*Il33*^Cit/+^. Mice in this facility tested positive for *Helicobacter* spp. Male and female age- and sex-matched mice were used and were age 6–12 weeks at the start of the experiments. The study was carried out following recommendations in the Guide for the Care and Use of Laboratory Animals of the National Institutes of Health. The CCHMC Institutional Animal Care and Use Committee approved the protocol. This study was carried out in accordance to ARRIVE guidelines.

### DSS-induced colonic injury and histopathologic examination

DSS (Colitis grade, GoJira Chemicals) was administered in the drinking water as a 2% (w/v) solution for up to 7 days to induce acute colitis^40^. Colons were stained with H&E and examined and histologically scored by light microscopy. DSS-induced colitis was scored by a blinded scorer on a 0–21 scale including: percentage of involved area, amount of follicles, edema, erosion/ulceration, crypt loss and infiltration of immune cells^40^. *Il10*^−/−^ colitis was scored by a blinded scorer on a 0–4 scale for each colon segment as previously described^41^.

### Immunofluorescence

For immunofluorescence analysis, frozen sections from *Il33*^*Cit/*+^ mice were fixed in 10% acetone for 15 min, rinsed in PBS, blocked with 3% donkey serum/PBS for 2 h at room temperature as previously described^18^, and incubated with primary Ab rabbit anti-mouse alpha smooth muscle actin (1:100, ab5694 from AbCam) or Alexa Fluor 594 anti-Vimentin Antibody (1:100, Biolegend, clone W16220A) in 3% normal donkey serum/PBS. For vimentin staining from WT and *Il1r1*^−/−^ mice, formalin-fixed paraffin-embedded sections were deparaffinized and rehydrated. Antigen retrieval was performed using citrate-based antigen unmasking solution (Vector Laboratories) for 10 min in a pressure cooker. Sections were rinsed in PBS, blocked with 3% donkey serum/PBS for 2 h at room temperature and incubated with primary Ab rabbit anti-Vimentin Antibody (ab92547, AbCam). After an overnight incubation at 4 °C, sections were washed with 0.1% BSA and 0.05% Tween/PBS and incubated with donkey anti-rabbit Alexa Fluor 594 (Invitrogen, Carlsbad, CA) for 2 h at room temperature. Slides were washed in PBS and counterstained with DAPI/Supermount G solution (Southern Biotechnology Associates, Birmingham, AL). Images were acquired using an Olympus BX51 microscope with a DP80 camera (Olympus America Inc., PA, USA) and CellSens Dimension digital imaging software (Olympus Corporation, version 1.18). Images were merged using ImageJ 1.52q (FIJI) software (NIH, https://imagej.nih.gov/ij/).

### RNA expression

RNA was isolated from tissue or cells using the RNeasy Mini Kit (Qiagen, Valencia, CA) per the manufacturer’s instructions, and RNA (100 ng) was reverse transcribed using the High-Capacity cDNA Reverse Transcription Kit (ThermoFisher Scientific, Waltham, MA) as previously described^18^. Real-time RT-PCR was performed with TaqMan Gene Expression Assays for *Il33* (Mm00505399), *Il1b* (Mm00434228_m1), *Tnf* (Mm00443258), *Il17a* (Mm01189488),* Ifng* (Mm01168134) and *Gapdh* (Mm99999915_g1). All reactions were performed on a StepOnePlus real-time PCR system (ThermoFisher Scientific). Relative mRNA levels were determined using the 2^−ΔΔCT^ method with *Gapdh* as the reference.

### Flow cytometric analysis and cell sorting

Mouse colons were washed in cold CMF-HBSS and placed in CMF-HBSS containing 5 mM EDTA and shaken gently at 37 °C for 30 min to remove epithelial cells. The remaining colon tissue was minced and agitated in RMPI with 2 mg/mL dispase (Gibco), 0.5 mg/mL collagenase I (Gibco) and 0.2 mg/mL DNase (Roche) at 37 °C for 45 min. Tissue was broken up using a 19-gauge needle and filtered through a 100 μM filter. Cells were centrifuged at 350 × *g* for 20 min and used for flow cytometry. For cell sorting, cells were resuspended in 40%Percoll/RPMI and spun at room temperature for 20 min at 600 × *g* and the cell pellet was collected. Single-cell suspensions were washed with FACS buffer (PBS/1% FCS) and incubated with combinations of the following Abs: APC-Cy7 anti-CD45 (Clone 30-F11), Pacific Blue anti-CD90 (Clone 30.H12), APC anti-EpCam (Clone G8.8), Alexa Fluor 594 anti-vimentin (Clone W1622A), APC anti-CD140a (Clone APA5), Alexa Fluor 647 anti-CD74 (Clone In1/CD74), PE anti-CD49a (Clone HMα1), PE Cy7 anti-F4/80 (Clone BM8) and PerCP Cy5.5 anti-CD3 (Clone 17A2) (Biolegend, San Diego, CA). Cells were then analyzed with an LSR Fortessa cytometer (BD Biosciences, San Jose, CA). Citrine^+^ cells were sorted with a Sony SH800S supported by NIH S10OD023410.

### In vitro and ex vivo colon fibroblast stimulation

WEHI-YH2 cells, a colon myofibroblast cell line^30^, or citrine^+^ cells sorted from DSS-treated mice were stimulated in vitro with 20 ng/mL IL-1β (Biolegend, San Diego, CA) for 18 h and then cells were harvested for RNA.

### Statistical analysis

For all data from experiments with three or more groups, non-parametric Kruskal–Wallis test was performed followed by two-stage step-up method of Benjamini, Krieger, and Yekutieli for false discovery rate. Data from experiments with two groups was analyzed using the non-parametric Mann–Whitney test. Rates of prolapse were compared using the Log-rank (Mantel-Cox) test. Spearman correlation was used for correlation analyses. Individual data points and mean ± SE are plotted on all graphs. The analysis was performed on Prism software (version 8.0.1, GraphPad Software, La Jolla, CA, www.graphpad.com). All authors had access to the study data and had reviewed and approved the final manuscript.

## Supplementary Information

Supplementary Figure 1.

## Data Availability

Data sharing is not applicable to this article as no datasets were generated or analyzed during the current study.
