# Supplementary figures and images for: IL-33 is produced by colon fibroblasts and differentially regulated in acute and chronic murine colitis
Source: Sci Rep. 2021 May 5;11:9575. doi: 10.1038/s41598-021-89119-1 (PMC8100152; doi:10.1038/s41598-021-89119-1)

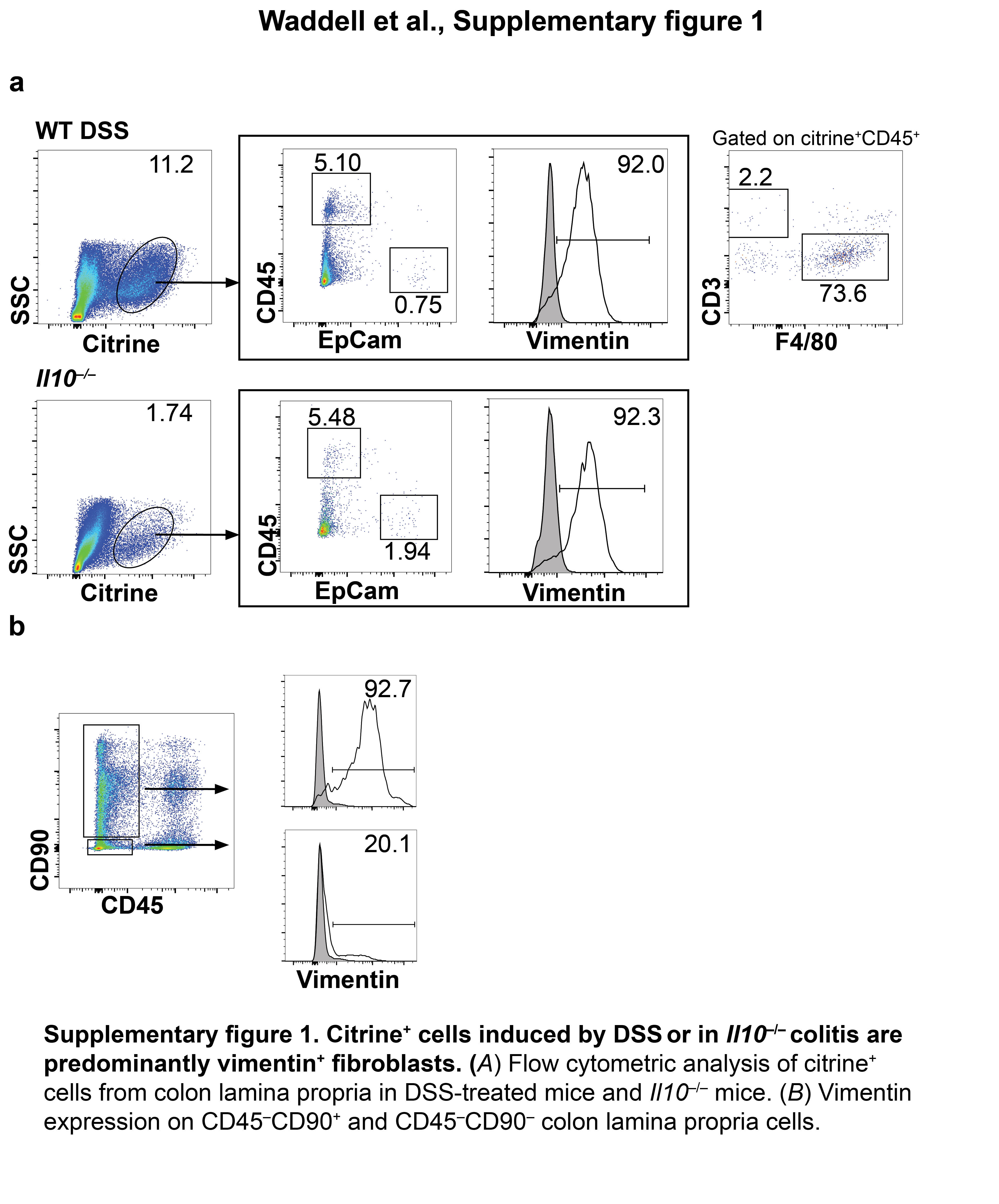

Supplement: Supplementary file 1 — Supplementary Figure 1. [file 41598_2021_89119_MOESM1_ESM.jpg]
